# Time-Dependent Dispersion of TiO_2_ Nanoparticles in 3D-Printed Denture Base Resin Using Probe Sonication

**DOI:** 10.3390/polym18192407

**Published:** 2026-10-02

**Authors:** Berivan Nawzad Hadi, Rizgar Mohammed Ameen Hasan

**Affiliations:** Department of Prosthodontics, College of Dentistry, Hawler Medical University, Erbil 44001, Iraq; rizgar.hasan@hmu.edu.krd

**Keywords:** titanium dioxide nanoparticles, probe sonication, sonication time, nanoparticle dispersion, 3D-printed denture base resin, PMMA, agglomeration

## Abstract

Titanium dioxide (TiO_2_) nanoparticles (NPs) can improve the performance of 3D-printed denture base resins; however, the reinforcing benefit depends on achieving uniform dispersion, and the effect of probe-sonication time on NP dispersion remains unclear. This study aimed to determine the sonication time (among 3, 5, and 7 min) producing the most qualitatively uniform TiO_2_ distribution. TiO_2_ NPs were silanized using 3-(trimethoxysilyl)propyl methacrylate, incorporated at 0.3 wt.% into a light-polymerizing 3D-printing resin, and probe sonicated. The resulting specimens were characterized by scanning electron microscopy, energy-dispersive X-ray spectroscopy, and Fourier transform infrared (FTIR) spectroscopy, and tested for surface roughness (Ra, μm) and Vickers hardness. Qualitatively, increasing sonication time progressively improved dispersion: the 7 min group showed the most homogeneous microstructure and titanium map. FTIR spectra were comparable across the groups, indicating preserved matrix chemistry. Surface roughness and hardness were not significantly different among the groups (*p* = 0.686 and *p* = 0.510, respectively). The 7 min group had the most qualitatively uniform TiO_2_ distribution. Although it had the numerically lowest surface hardness, it was not comparatively statistically significant. Therefore, within the tested conditions, the 7 min can be described as the most suitable duration for the primary dispersion objective. Additional tests (flexural strength, flexural modulus, and impact toughness) and degree-of-conversion analysis are necessary to confirm the effects of different sonication times.

## 1. Introduction

Polymethyl methacrylate (PMMA) has been the most common material used in denture bases since the 1940s, primarily due to favorable handling and esthetic attributes and the ability to fabricate and repair the material clinically. Additionally, although PMMA is described as biocompatible, it is prone to microbial adhesion that can contribute to discoloration and biological complications such as denture-stomatitis. It also exhibits a number of other unfavorable characteristics such as low fracture resistance, low mechanical strength (including poor resistance to fatigue, impact, and bending) and low wear resistance, alongside polymerization shrinkage [1,2,3].

In another area of dentistry, digital manufacturing has reshaped denture base fabrication via computer-aided design/computer-aided manufacturing (CAD-CAM) through either subtractive or additive methods, such as, milling or three-dimensional (3D) printing, respectively [4]. Although 3D-printed resins offer numerous advantages such as improved accuracy, reduced material waste, and efficient fabrication, concerns still remain regarding their mechanical properties and long-term durability when compared with conventional PMMA materials [5]. For instance, multiple reports indicate that 3D-printed denture base resins can exhibit significantly lower microhardness, flexural strength, and modulus compared with other denture base resins, and broader syntheses conclude that 3D-printed denture bases have inferior mechanical properties compared with milled alternatives while also highlighting heterogeneity driven by differences in materials, printing orientation, and post-curing protocols [4,6].

Consequently, given these mechanical and biological limitations and the increasing adoption of 3D printing in prosthodontics, there is a growing need to investigate the modification of 3D-printed denture base resins, particularly through the use of nanoparticles (NP)s, to improve their mechanical properties, antimicrobial properties, and clinical performance [3,7].

Titanium dioxide (TiO_2_) NPs are repeatedly highlighted as a candidate. They are described as non-toxic, chemically inert, with high hardness and antibacterial activity; it has been reported that the antimicrobial properties are inherent and act through the production of cytotoxic oxygen radicals that can provide antimicrobial effects when incorporated into PMMA denture base material systems [3,7]. Beyond denture-base PMMA, TiO_2_ NPs are additionally described as multifunctional dental polymer additives whose photoactivities and contributions to mechanical properties can improve resin performance, including improvements in degree of conversion, elastic modulus, and hardness [8].

NP-filled polymers are highly sensitive to agglomeration and dispersion state; however, achieving a uniform dispersion of NPs within the resin matrix is central to obtain their advantageous qualities, as NP agglomeration can negatively affect the material’s mechanical properties and overall performance [9,10]. NP powders can form agglomerates upon wetting due to attraction forces such as van der Waals forces and liquid surface tension. Dispersion and deagglomeration are therefore emphasized as necessary to obtain the full benefits of the particles—particularly for NPs whose characteristics are described as exploitable only in a well-dispersed state [10]. Empirically, electron microscopy observations reveal that NPs may persist as aggregates rather than dispersing individually, and dispersion efficiency has been reported as a determining aspect of mechanical performance in NP–polymer systems [9]. Additionally, dispersion outcomes can depend on polymerization conditions; slower polymerization is reported to promote large-length-scale network structures while faster polymerization yields better NP dispersion, highlighting the need to control processing to prevent states that can compromise functional performance [11].

Probe sonication is widely used to obtain and maintain NP suspensions, and has been employed to disperse NPs in conventional denture base systems, particularly in PMMA monomer prior to polymerization, with typical sonication durations of approximately 3 min reported to achieve adequate NP distribution [12]. This is because the alternating high and low-pressure cycles generate cavitation that provides energy for disrupting agglomerates [13]. In comparison with other dispersion technologies, ultrasound has been reported to offer considerable advantage for dispersing NP agglomerates, yielding smaller particles and increased size uniformity in general, while dispersion effectiveness depends on multiple factors such as power, time, frequency, ultrasonicator type, and suspension properties (e.g., viscosity, temperature, and particle concentration) [14,15].

Sonication time is a key variable because it has been shown to significantly affect resulting material characteristics, with structural and chemical characterization reported to track the effects of sonication time in nanocomposite films [16]. Evidence indicates the presence of an optimum duration, where tensile strength and fracture toughness initially increase with sonication time but subsequently decrease at longer sonication times, and separate work indicates that dispersion is most effective in the early stage of sonication and becomes more difficult to improve later, supporting the concept that more sonication is not necessarily better [17,18]. Additionally, overly lengthy sonication times can also be detrimental, with reports of nanofiller damage, poor distribution, and re-agglomeration mechanisms, reinforcing the need for time optimization [19].

For TiO_2_ suspensions specifically, one study reports that 20 min of ultrasonication at 180 W is sufficient for dispersing a rutile TiO_2_ NP suspension stable for more than 1 h, while also emphasizing that the suitable sonication time is influenced by factors including NP concentration, solution chemistry, and sonicator parameters [20]. In light of (i) inferior mechanical performance reported for 3D-printed denture base resins relative to milled materials, (ii) established mechanical and antimicrobial benefits reported for TiO_2_ incorporation in PMMA and dental resin systems, (iii) the strong dependence of NP-enabled properties on dispersion and sonication parameters, and (iv) the lack of extensive investigations of these methods for liquid 3D-printed denture base resins (which differ in composition and viscosity from conventional PMMA) [3,4,9,21]. This study intended to investigate the effect of sonication time on the controlled dispersion of TiO_2_ in 3D-printed denture base resin at the same TiO_2_ content and sonication conditions and the influence of such dispersion on surface characteristics. The TiO_2_ dispersion was characterized using SEM and EDS‚ the chemical integrity of the matrix was evaluated using ATR-FTIR‚ and the roughness and Vickers microhardness were all evaluated in detail. It was hypothesized that longer sonication time would improve TiO_2_ dispersion‚ although it would influence hardness in an unknown way‚ as better dispersion could give better reinforcement‚ or on the contrary‚ due to the more energy input to reach better dispersion‚ reduce the topography of the matrix after curing.

## 2. Materials and Methods

Commercial TiO_2_ NPs (rutile phase, near-spherical morphology, ≥99.9% purity; US Research Nanomaterials, Inc., Houston, TX, USA) were used, which have an average particle size of 50 nm and a specific surface area of 20–40 m^2^/g, as reported by the manufacturer.

### 2.1. Silanization of TiO_2_ NPs

The TiO_2_ surface was priorly treated with a silane coupling agent—3-(trimethoxysilyl)propyl methacrylate (98%; Thermo Fisher Scientific‚ Fair Lawn‚ NJ‚ USA)—to improve the interfacial adhesion of the TiO_2_ NPs to the resin matrix.

The silanization procedure followed the protocol reported by Nguyen et al. [22]. The silane quantity was calculated according to the formula (Equation (1)) provided by Chaijareenont et al. [23]

(1) $$\mathrm{Silane}\left(g\right)=\frac{\mathrm{Filler}\left(g\right)\times \mathrm{Specific}\mathrm{Surface}\mathrm{Area}\left(\frac{m^{2}}{g}\right)}{\mathrm{Minimum}\mathrm{Coating}\mathrm{Area}\mathrm{of}\mathrm{Silane}\left(\frac{m^{2}}{g}\right)}$$

where the *minimum coating area* was considered as 314 m^2^/g [23]. The TiO_2_ *filler* and *specific surface area* chosen for the equation were 10 g and 30 m^2^/g, respectively.

3-(trimethoxysilyl) propyl methacrylate silane (0.955 g, corresponding to 9.55 wt.% relative to TiO_2_; ~0.91 mL) was initially dissolved in acetone (80 mL). The TiO_2_ NPs were then dispersed in the above-mentioned silane solution and continuously stirred (800 rpm) for 1 h at 50 °C to allow the interaction of the molecules of the silane with the NP surface. The solvent was then removed with an oven evaporator at 100 °C for 120 min, and the silanized powder was dried at 100 °C for 6 h. Finally, the silanized TiO_2_ NPs were allowed to cool to room temperature before use.

### 2.2. Preparation of TiO_2_-Modified Denture Base Resin

The silanized TiO_2_ NPs were weighed and mixed into a light-polymerizing denture base resin (IMPRIMO LC Denture 385 nm‚ SCHEU-DENTAL GmbH‚ Iserlohn‚ Germany) at 0.3 wt%‚ based on 100 mg of total mass of the resin. This wt% was decided based on Vicker’s hardness and surface roughness results of a prior pilot study comparing different low wt% (0.05, 0.1, 0.2, 0.3, 0.4) TiO_2_-incorporation into 3D printing resin.

The mixture was manually stirred until a homogeneous mix was visually confirmed and then subjected to probe sonication with an ultrasonic cell processor (Model UH-950W, Tianjin Automatic Science Instrument Co., Ltd., Tianjin, China; 950 W, 220 V, 50 Hz, 10 mm probe; manufacturer not specified) in pulsed mode for a total time of either 3, 5, or 7 min using alternating 2 s sonication and 2 s pause intervals (2 s on/2 s off; 50% duty cycle) at 150 W (or ~16% of sonicator power) to disperse the TiO_2_ NPs and reduce agglomeration.

Additionally, to prevent overheating and premature polymerization of the resin‚ the mixing vessel was kept in an ice bath during the sonication. The irradiation times of 3‚ 5 and 7 min were used to evaluate the effect of sonication on the dispersion of the NPs‚ while other operating parameters were kept constant.

### 2.3. Specimen Fabrication

Disc-shaped specimens (10 mm × 2 mm) were designed using the open-source CAD software Asiga Composer (version 1.3‚ 2021; Asiga‚ Sydney‚ Australia)‚ then exported as STL files before additive DLP polymerization using the IMPRIMO 3D printing system and IMPRIMO LC Denture resin (385 nm; SCHEU-DENTAL GmbH‚ Iserlohn‚ Germany). The STL files were imported into Print Setup (SprintRay Inc., Los Angeles, CA, USA; available online: [https://sprintray.com/software/], accessed on 2 July 2026), where the samples were aligned parallel to the nominal build direction (0° orientation) for consistency; minimizing geometric distortion and ensuring reproducibility. A layer resolution of 0.05 mm (50 µm) was favorably selected to balance surface fidelity with fabrication time. Support structures were automatically generated by the slicing software and refined manually to reduce stress-concentration points; strategically placing them on non-critical surfaces to avoid interference with mechanical testing regions and balance them with ease of detachment during finishing. The printer was calibrated according to manufacturer instructions, ensuring proper leveling and projector focus. The 405 nm UV projector cured entire layers simultaneously at a light intensity setting of 6 mW/cm^2^; each layer was exposed for 2.5 s to achieve full polymerization while minimizing thermal stress. After printing, the build platform lifted the samples vertically from the resin vat. The samples were separated using a precision scraper to avoid mechanical damage.

The specimens were then washed in an ultrasonic bath containing 99% IPA for 5 min, followed by air drying. Post-curing was performed in a SprintRay Pro Cure unit (SprintRay Inc., Los Angeles, CA, USA) at 60 °C for 20 min. The post-curing parameters are reported as applied experimentally. The support structures were meticulously removed using hand rotary tools, and surfaces were polished under precooled water with 600-grit and 1200-grit silicon carbide papers.

The total number of specimens fabricated was 45, nine samples for characterization studies, and eighteen samples for each of hardness and surface roughness tests.

### 2.4. Characterization of NP Dispersion

The dispersion and incorporation of the TiO_2_ NPs into the 3D-printed denture base resin matrix were evaluated by SEM to observe the resin surface morphology‚ energy-dispersive X-ray spectroscopy (EDS) to assure the incorporation of TiO_2_ into the resin matrix‚ and attenuated total reflectance Fourier-transform infrared (ATR-FTIR) spectroscopy to confirm the interaction between the NPs and the resin matrix. All of these analyses were conducted to investigate the suitable time period of sonication corresponding to the uniform dispersion of TiO_2_ NPs.

#### 2.4.1. SEM

Surface morphology of the NPs was observed for qualitative assessment using an SEM device (Quanta 450‚ FEI Company‚ Hillsboro‚ OR‚ USA). Before imaging‚ the samples were mounted on conductive copper stubs using double-sided carbon tape and sputtered with a thin layer of gold [24]. The gold layer helped increase conductivity and minimize charging of the samples under electron beam exposure. The samples were imaged at high vacuum using an optimized accelerating voltage and beam current for high-resolution imaging of polymer nanocomposites. Micrographs were taken at different magnifications to determine the dispersion of the titania and the morphology of the individual TiO_2_ agglomerates.

#### 2.4.2. EDS

The elemental composition and the distribution of the TiO_2_ NPs within the resin matrix were analyzed by an EDS device (XFlash 5010, Bruker Nano GmbH‚ Berlin‚ Germany) coupled with the SEM device. Elemental mapping was obtained to observe the distribution of element constituents of the denture base resin and of titanium (Ti). Mapping the signals of all the elements gives their spatial distribution. A uniform Ti signal would indicate a successful chemical incorporation of the NPs into the matrix and a localized or clustered Ti signal would indicate agglomeration possibly due to the formation of a weak or optically inhomogeneous zone [25].

#### 2.4.3. ATR-FTIR

The chemical structure and possible interaction between the silanized TiO_2_ NPs and the denture base resin were studied using an ATR-FTIR spectrophotometer (FT/IR-4600; JASCO‚ Tokyo‚ Japan) in absorbance mode. The FTIR spectrum in the 4000–400 cm^−1^ wavenumber range was obtained at a resolution of 4 cm^−1^‚ with an average of 32 scans to improve the signal-to-noise ratio [26].

The resulting spectra were examined to identify bands attributed to the polymer matrix and TiO_2_ NP incorporation. Attention was paid particularly to the shifting of bands‚ intensity changes and broadening of bands‚ as these are indicative of possible physicochemical interaction between NPs and polymer matrix. Comparison of neat resin spectra with TiO_2_-modified resin spectra allows differentiation between physical incorporation and chemical interaction between NPs and resin matrix.

### 2.5. Surface and Mechanical Characterization

#### 2.5.1. Surface Roughness

Surface roughness was assessed via arithmetical mean roughness (Ra, μm) using a portable stylus profilometer (TIME 3200/TR200, Beijing TIME High Technology Ltd., Beijing, China). The instrument operated at a cutoff length of 0.8 mm, an evaluation length of five cutoff lengths (4.0 mm), and with an RC filter. Three different points were scanned for each specimen. Six specimens per group were tested. Finally, the averaged surface roughness (Ra, μm) for each specimen and group was calculated.

#### 2.5.2. Hardness Test

Vickers hardness test (HV0.3) was performed using a GT-TEST Vickers hardness tester (Golden Time Technology Development Limited, Chengde, Hebei, China). Six specimens were tested. Three indentations were performed per specimen, and the mean hardness value was calculated.

A polished sample was placed under the hardness tester, a pyramidal diamond indenter was pressed into the material under a test load of 0.3 kgf for 15 s dwell time. The size of the indentation was measured under a microscope, hardness value was calculated from the applied force and the indentation diagonal lengths.

### 2.6. Generative Artificial Intelligence Use in Manuscript Preparation

Generative artificial intelligence (GenAI) tools were used in the preparation of this manuscript. Elicit AI (web-based AI research assistant; Elicit Research PBC, Covina, CA, USA; available online: [https://elicit.com/], accessed on 4 August 2026) was used to aid in literature discovery and the identification of relevant publications. ChatGPT (GPT-5.6 Sol; OpenAI, San Francisco, CA, USA) was used as an assisting tool for the writing and editing of parts of the article‚ and assistance in the interpretation‚ discussion of FTIR‚ EDS and SEM data. All the outputs provided by the ChatGPT API were reviewed by the authors‚ verified against the original data and cited literature‚ and revised. The authors take full responsibility for the accuracy, interpretation, and conclusions presented in this work.

## 3. Results

### 3.1. SEM Analysis

**Figure 1:**
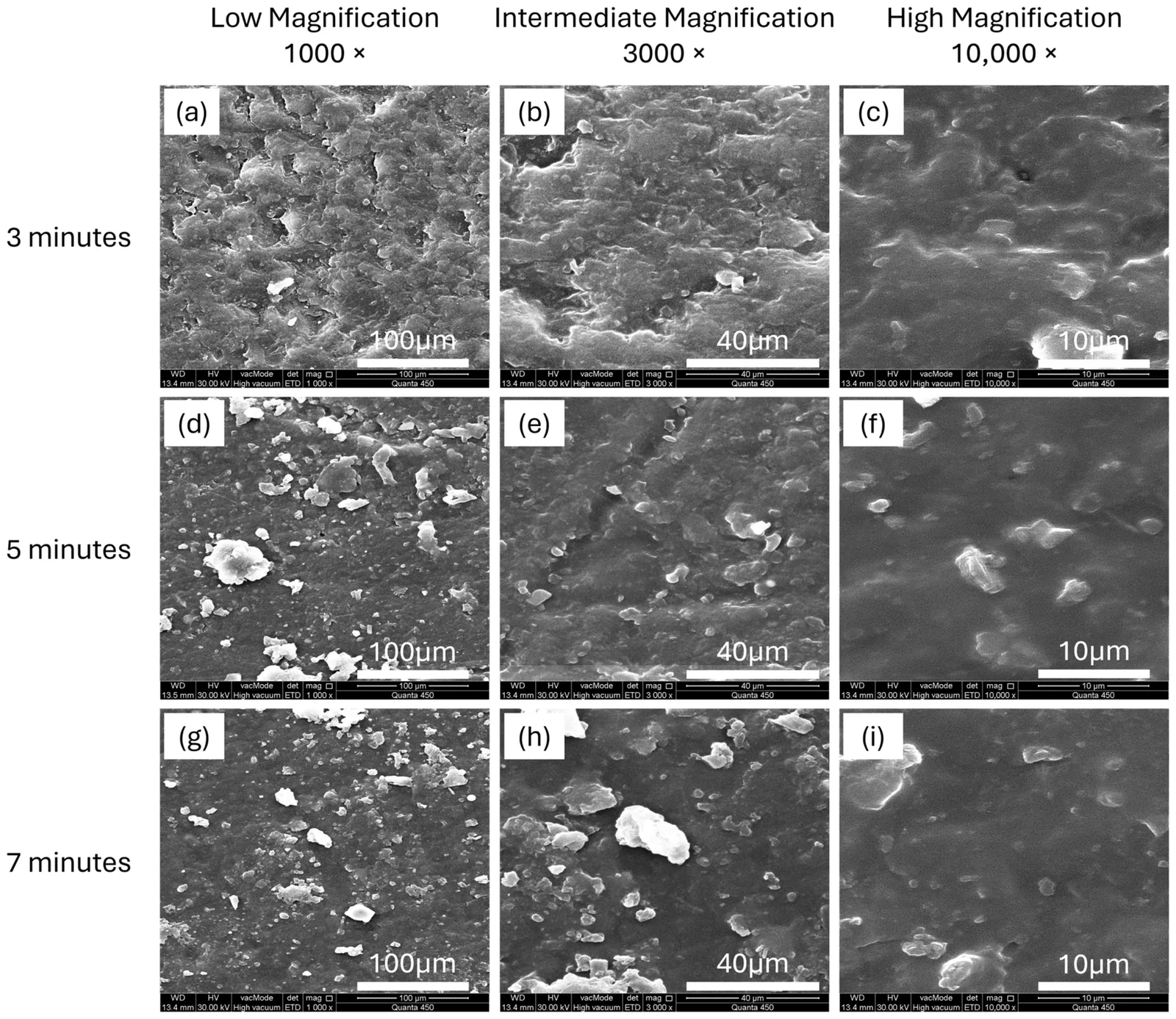
SEM micrographs of 3D-printed titanium dioxide (TiO_2_)-incorporated resin subjected to probe sonication for 3 min (**a**–**c**), 5 min (**d**–**f**), and 7 min (**g**–**i**). Images were acquired at 1000× (**a**,**d**,**g**), 3000× (**b**,**e**,**h**), and 10,000× (**c**,**f**,**i**) magnification, with corresponding scale bars of 100, 40, and 10 µm, respectively. The micrographs illustrate differences in surface morphology and the apparent distribution and agglomeration of the incorporated particulate phase as a function of sonication time.

The dispersion of TiO_2_ NPs in the denture base resin matrix of the 3D-printed samples of the three groups (3‚ 5, and 7 min sonication) was qualitatively evaluated in the SEM images shown in Figure 1. The representative electron micrographs of the three groups at three different magnifications (3 min (a–c)‚ 5 min (d–f) and 7 min (g–i)) are displayed. The differences between distributions of particles and agglomerates appeared in micrographs with increased sonication time.

The surface of the resin appeared rough and matrix dominated‚ with some local hollows‚ small porosities‚ and few protruding particles in the 3 min group (Figure 1a–c). NPs were observed enclosed in the matrix‚ but also dispersed in the matrix‚ which can be seen by the presence of some bright areas (potential local aggregates). However‚ higher magnifications indicate that the individual particles had fused into micron-sized aggregates, suggesting insufficient ultrasonic energy.

In the 5 min group (Figure 1d–f)‚ the discrete particles appear more dispersed on the surface of the resin and more spread out than in the 3 min group. Clusters of high particle concentrations‚ including several large point-like micron-sized agglomerates‚ were still observable but generally smaller than those in the 3 min group. However, they were distinctly observed on the resin surface‚ indicating a partial improvement in dispersion.

The particulates (Figure 1g–i) were most evenly dispersed in the 7 min group‚ where the distribution of smaller features in the resin was broader. At high magnification‚ small agglomerates were less frequent‚ while large isolated agglomerates still remained. The results suggest that the 7 min sonication time allowed for a qualitatively better dispersion of particulates‚ without complete deagglomeration.

**Figure 2:**
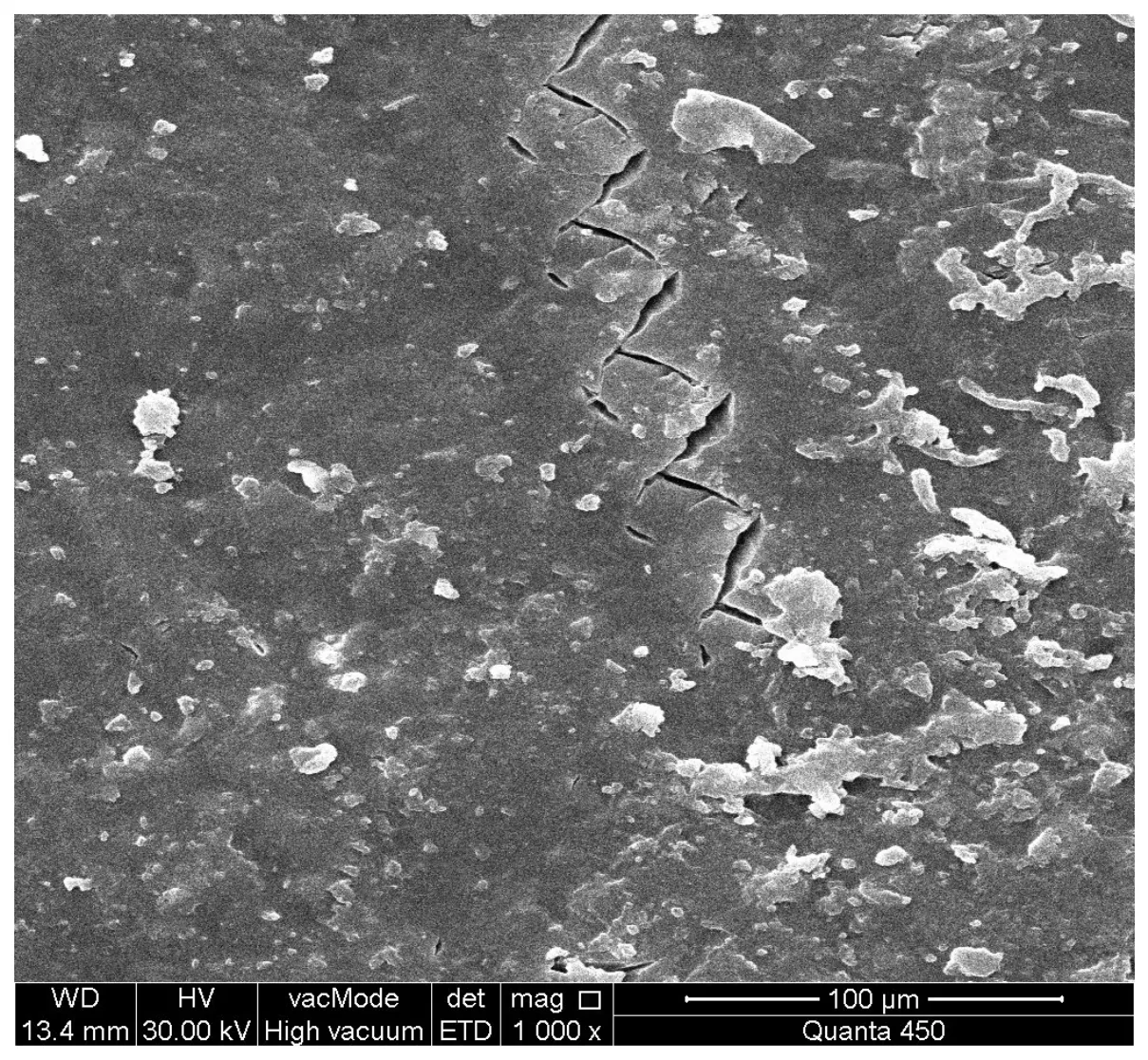
SEM image of the 7 min sonicated specimen at 1000× magnification showing a branched microcrack.

In addition, a branched microcrack was observed in one of the examined fields of the 7 min group, which was roughly 5 µm wide (Figure 2). These were not observed in the 3 min and 5 min sonication groups.

Qualitatively, the highest agglomeration was observed in the 3 min sample‚ whilst the 7 min sample showed the most even distribution. The increased probe-sonication time indicates reduced domain size and frequency of the surface agglomerates‚ leading to a more uniform microstructure. However, since SEM contrast and morphology alone cannot determine the composition of the particles‚ EDS mapping was used to confirm that the particulate matter was TiO_2_.

### 3.2. Elemental Distribution (EDS Analysis)

**Figure 3:**
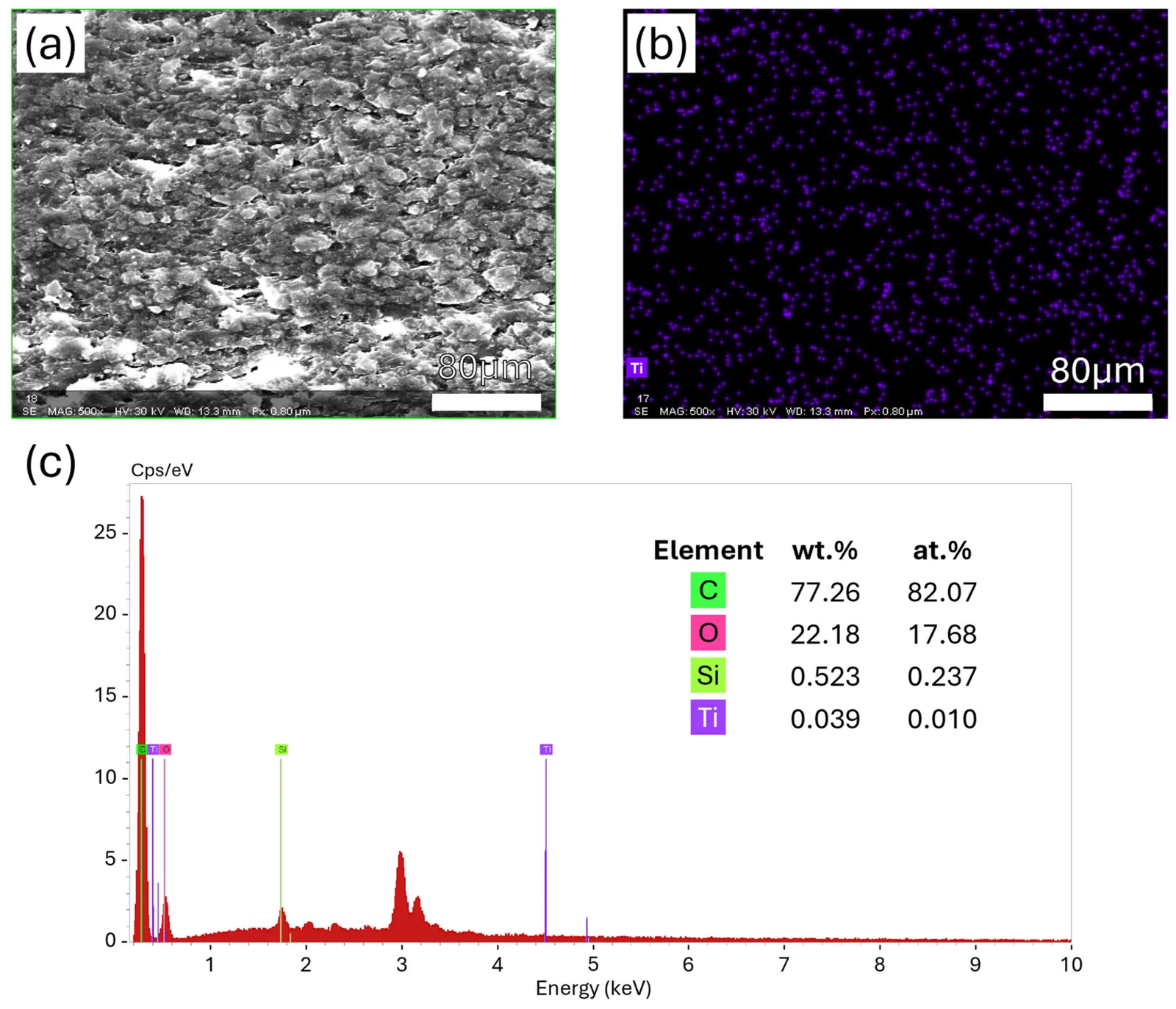
SEM-EDS analysis of the 3 min probe sonicated samples: (**a**) SE-SEM surface image showing particle distribution; (**b**) corresponding elemental map of titanium (Ti); and (**c**) EDS spectrum accompanied by the quantitative elemental composition table (wt% and at%).

**Figure 4:**
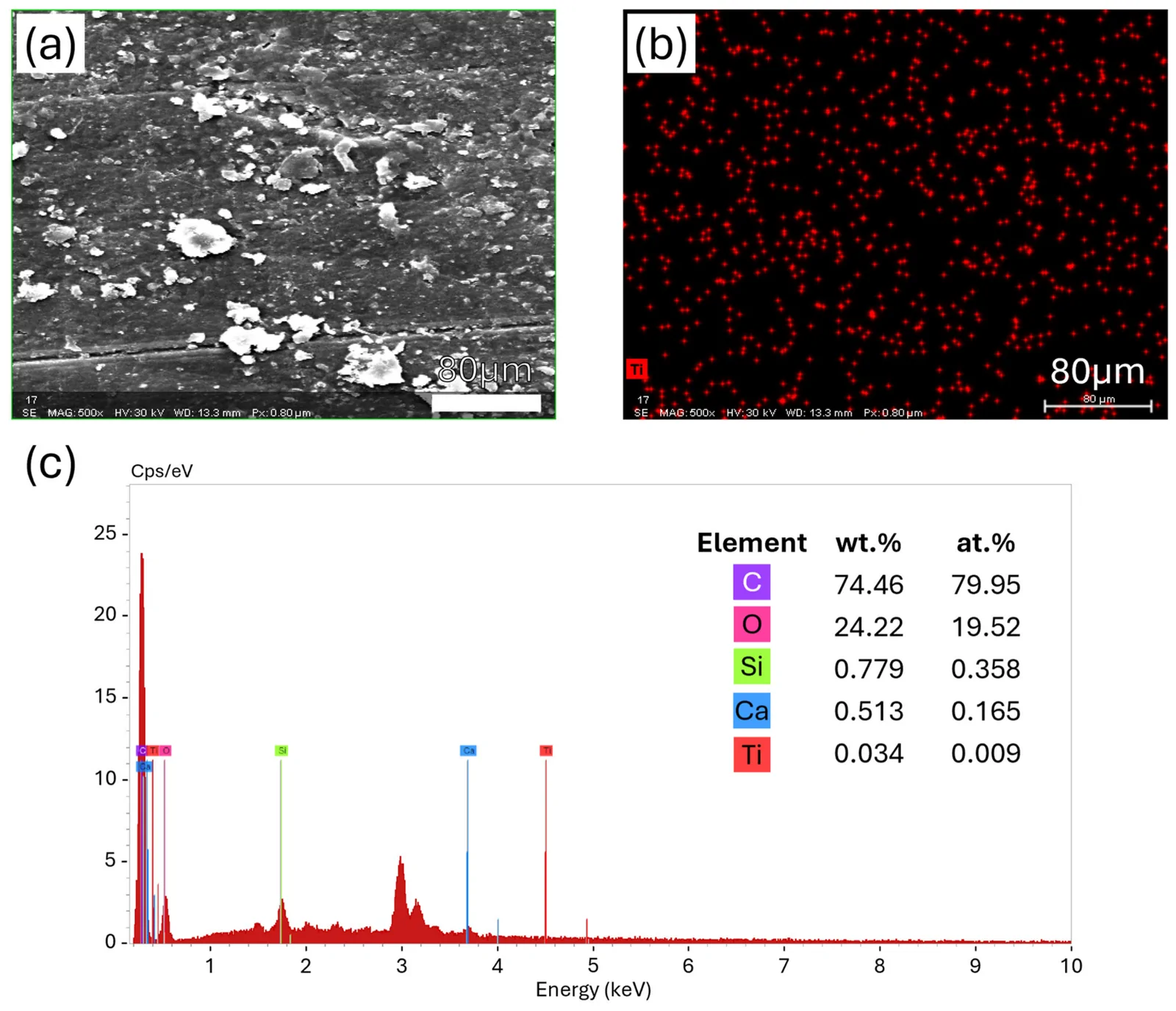
SEM-EDS analysis of the 5 min probe sonicated group: (**a**) SE-SEM surface morphology showing particle distribution; (**b**) corresponding elemental map of Ti; and (**c**) EDS spectrum accompanied by the quantitative elemental composition table (wt% and at%).

**Figure 5:**
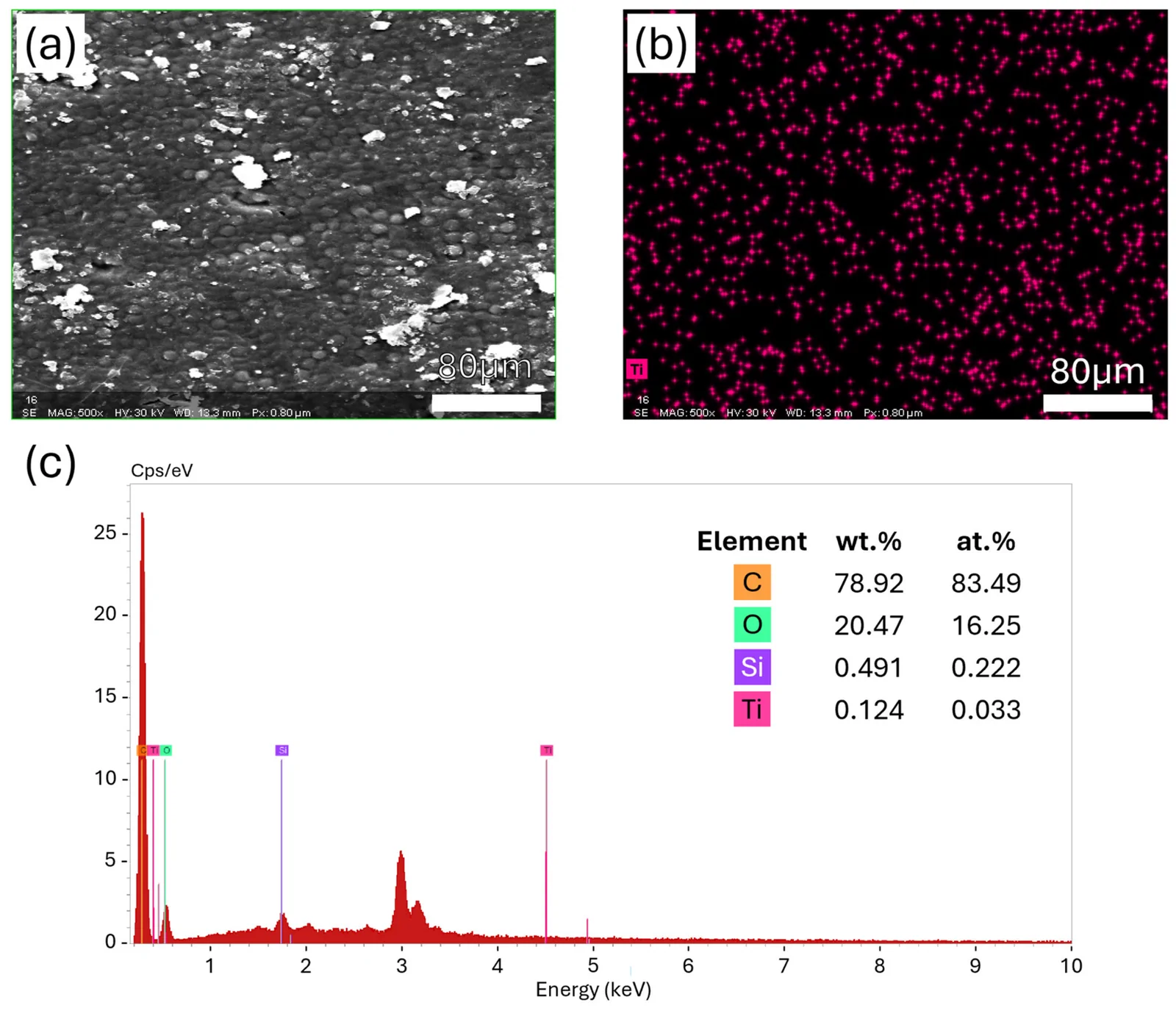
SEM-EDS analysis of the 7 min probe sonicated group: (**a**) SE-SEM surface morphology showing particle distribution; (**b**) corresponding elemental map of Ti; and (**c**) EDS spectrum accompanied by the quantitative elemental composition table (wt% and at%). Note the locally higher Ti signal, showing spatial heterogeneity within the analyzed area rather than a difference in total Ti content (nominal loading fixed at 0.3 wt.% in all groups).

The quantitative EDS analysis presented in Figure 3c, Figure 4c and Figure 5c, shows that C and O were the most abundant elements in all 3 groups‚ with Si present in minor amounts.

The SEM image and the corresponding Ti-map of the 3 min group are shown in Figure 3a and b, respectively. The surface morphology reveals a rough and heterogeneous surface topography with multiple bright areas improved with particles. The Ti-mapping shows the surface is populated with Ti-containing particles‚ but their distribution is heterogeneous, appearing as discrete particles or aggregates. This indicates that the mixing process over 3 min of sonication was sufficient to generate a homogeneous dispersion of TiO_2_ in the matrix‚ but some agglomerates and Ti-deficient regions persist.

The overall appearance in the 5 min group (Figure 4) is still quite rough and heterogeneous‚ with bright particles shaped as individual grains surrounded by areas of high particle density and some large aggregate structures with many smaller grains. The Ti map shows Ti is spread out across most of the area and concentrated in spots‚ but in discrete sizes. Probe sonication for 5 min concentrated the TiO_2_ in the matrix but did not completely deagglomerate the TiO_2_ in the nanocomposite.

Additionally, although the quantitative composition of Ti for the 5 min group (0.034 wt%, 0.009 at%) was similar to that of the 3 min group (0.039 wt%, 0.010 at%), as shown in Figure 3c and Figure 4c, these suggest heterogeneity in the spatial distribution of Ti in the analyzed fields. Trace amounts of calcium (<1 wt%) were detected only in the 5 min group, which can be attributed to environmental surface contamination during sample preparation.

Figure 5 depicts the EDS results of the 7 min group. In the SEM image (Figure 5a), the matrix appears textured and heterogeneous‚ with numerous bright particles spread throughout the matrix, which look less aggregated and more dispersed than the shorter duration treatments; however‚ some larger domains of particle-rich regions are still observed. The Ti map shows a similar signal level over the entire scanned area‚ with some areas free of Ti. Similar to the SEM analyses (Section 3.1.), the EDS results suggest that the prolonged sonication time has improved the dispersion and homogeneity of TiO_2_‚ while complete elimination of agglomeration was not obtained. Additionally, the 7 min group showed a higher locally detected Ti value (0.091 wt.%) compared with the 3 min and 5 min groups (see Figure 5c). However, since the nominal Ti loading was fixed at 0.3 wt.% for all groups and EDS provides localized, semi-quantitative elemental analysis, these differences should not be interpreted as variations in the overall Ti content. Rather, they qualitatively suggest heterogeneity in the spatial distribution of Ti within the analyzed areas.

### 3.3. Chemical Structure (ATR-FTIR)

**Figure 6:**
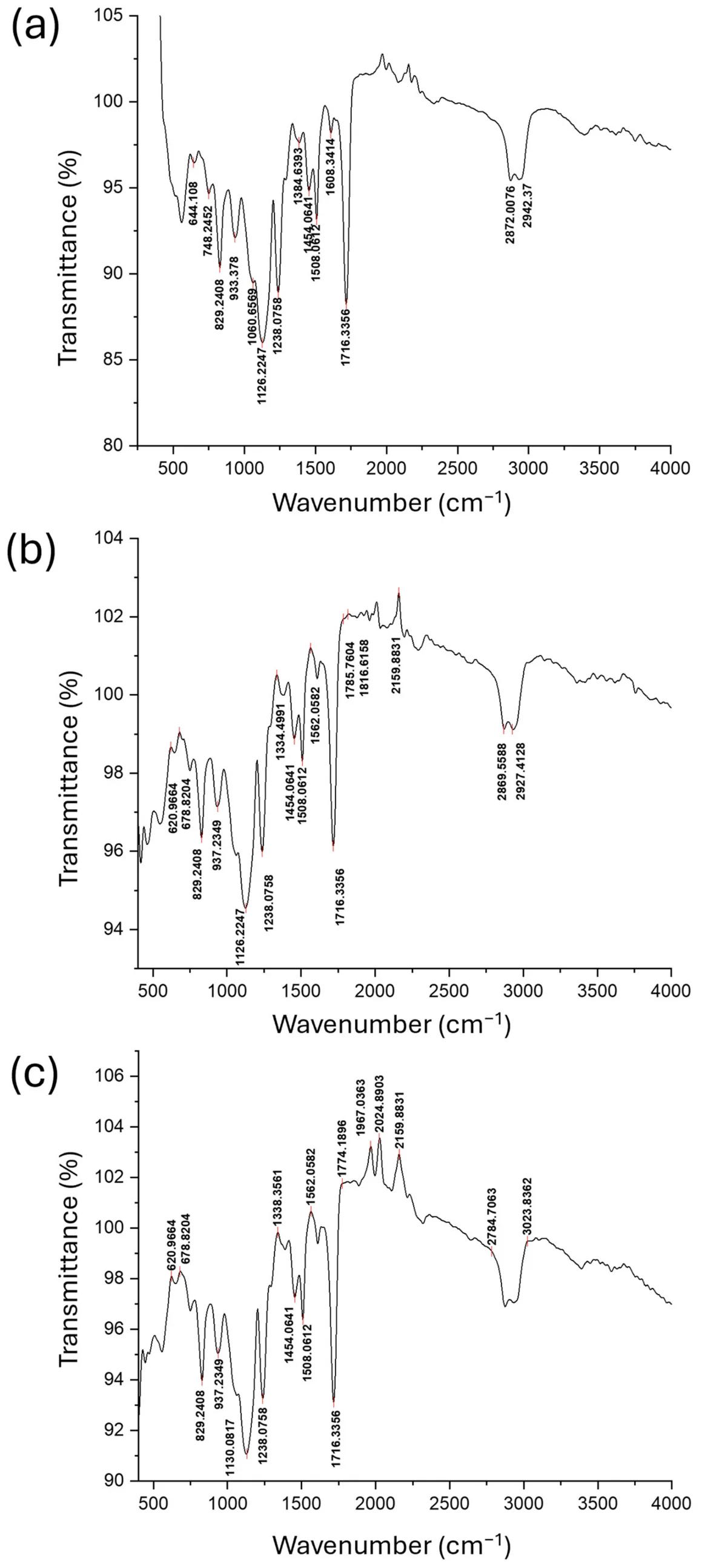
Fourier transform infrared (FTIR) spectroscopic characterization of TiO_2_-reinforced resin processed under varying probe sonication times: (**a**) 3 min, (**b**) 5 min, and (**c**) 7 min.

The FTIR spectra of all three groups retained the main absorption bands of the resin matrix‚ which indicates that the chemical structure was not altered with different sonication times (Figure 6). The spectra of each sample showed the spectral ranges of the aliphatic C-H stretching vibration (~2870–3020 cm^−1^)‚ the strong carbonyl C=O stretching vibration (~1716 cm^−1^)‚ the skeletal vibration and C-H deformation vibration of the matrix (~1330–1610 cm^−1^)‚ and the C-O/C-O-C stretching vibrations (~1060–1240 cm^−1^). Broad features at low wavenumbers near ~620–680 cm^−1^ (or <800 cm^−1^ in the 3 min spectrum) are attributed to vibrations involving Ti-O and Ti-O-Ti and are evidence of the addition of TiO_2_ into the structure. Weak features between ~1967 and 2160 cm^−1^ are present in the 7 min spectrum (Figure 6c), and can be overtone‚ combination‚ or weak background bands; these features need to be interpreted with caution.

### 3.4. Results and Statistical Analysis of Surface and Mechanical Characterization

The Ra and surface hardness of the specimens were analyzed statistically to observe differences between the three time groups using one-way ANOVA and post hoc pairwise comparison (Duncan’s multiple range test‚ *p* < 0.05).

The three treatment-time groups were formed from different experimental specimens, so the observations at 3, 5, and 7 min were treated as independent. For each specimen, the three technical readings were first averaged to obtain a single specimen-level value. The technical readings were therefore not counted as separate observations. Accordingly, six independent specimen-level observations were available at each treatment duration for both surface roughness and hardness (*n* = 6 per group).

Treatment-time differences were evaluated using a one-way analysis of variance (ANOVA). Model assumptions were assessed from the ANOVA residuals using the Shapiro–Wilk test and normal Q-Q plots for residual normality, and the mean-centered Levene test for homogeneity of variance. Internally studentized residuals were also examined for unusually large departures. Because these diagnostics did not show a clear violation that would invalidate the parametric comparison (see Table 1), conventional one-way ANOVA was retained as the primary analysis. Tukey’s HSD was planned only if the omnibus ANOVA was significant. Effect size was summarized by eta-squared (η^2^), and all tests were two-sided with *p* < 0.05 taken as statistically significant.

Given the small group size, Welch’s ANOVA was also calculated as a sensitivity analysis. This comparison does not require equal group variances and was used to check whether the substantive conclusions changed when the homogeneity assumption was relaxed.

For surface roughness, the residual Shapiro–Wilk test was not significant (W = 0.929, *p* = 0.186), and Levene’s test likewise did not reach significance (F = 3.194, *p* = 0.070). The largest absolute internally studentized residual was 2.69. For hardness, the residual distribution also showed no statistically significant departure from normality (W = 0.909, *p* = 0.083), while Levene’s test provided no evidence of unequal variances (F = 0.304, *p* = 0.742); the maximum absolute internally studentized residual was 1.59. The corresponding Q-Q plots are reported as Supplementary Figures S1 and S2.

#### 3.4.1. Descriptive Results

Table 2 summarizes the specimen-level distributions. Surface roughness was numerically highest at 5 min. Hardness was similar at 3 and 5 min and lower, on average, at 7 min. These differences were interpreted together with the within-group variability and the inferential results rather than from the means alone.

#### 3.4.2. Surface Roughness Results

**Figure 7:**
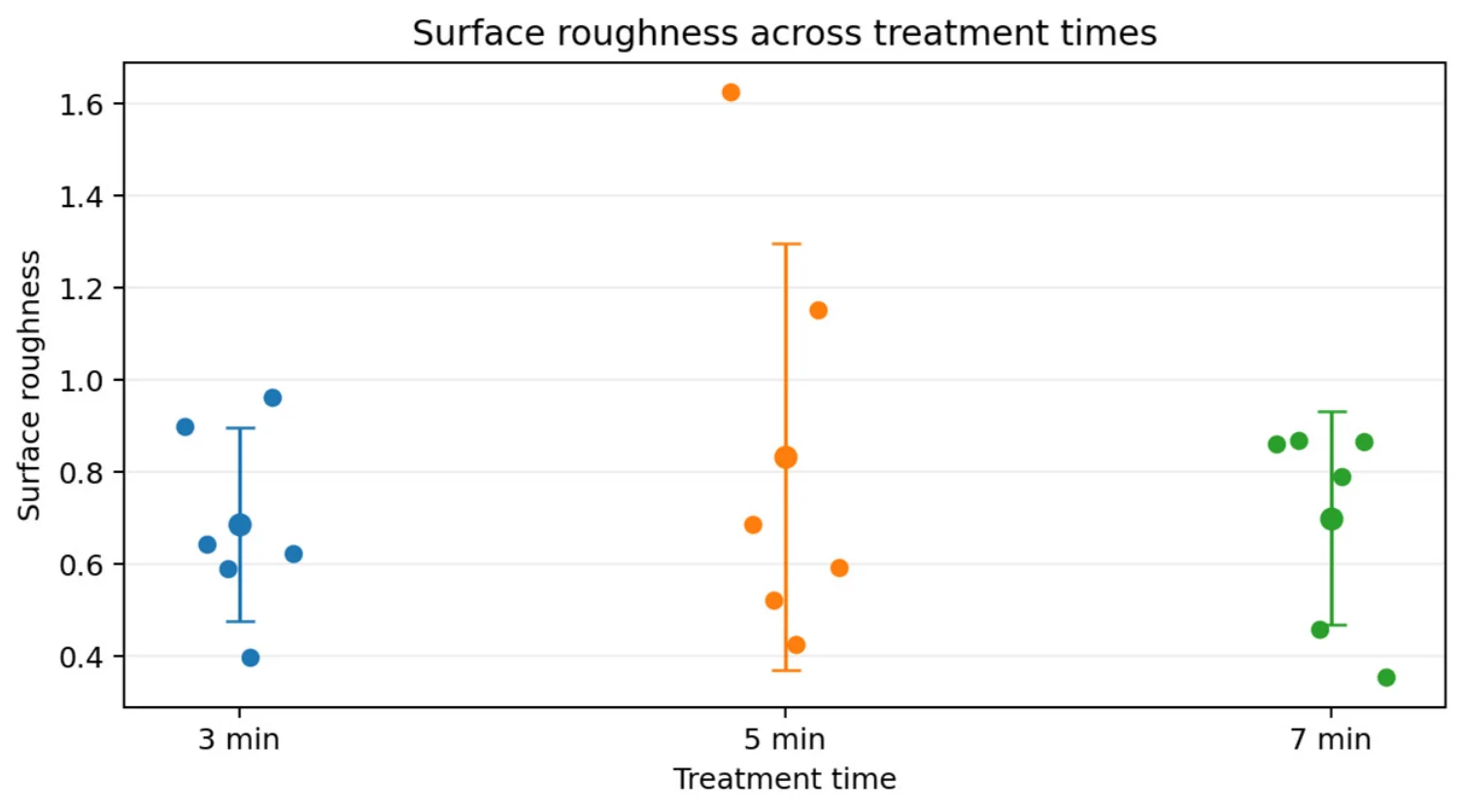
Surface roughness across different time intervals (3, 5, and 7 min). Points represent specimen-level values; the larger marker denotes the group mean; and error bars represent ± 1 standard deviation (SD).

Mean surface roughness was 0.687 ± 0.209 at 3 min, 0.835 ± 0.463 at 5 min, and 0.701 ± 0.231 at 7 min (see Figure 7). The one-way ANOVA showed no statistically significant treatment-time effect, F(2, 15) = 0.386, *p* = 0.686, η^2^ = 0.049. Thus, the higher mean observed at 5 min should be viewed as a numerical difference rather than evidence of a systematic increase. The pattern was also non-monotonic, with the 7 min mean returning close to the 3 min level.

The sensitivity analysis gave the same conclusion. Welch’s ANOVA was non-significant, F(2, 9.40) = 0.242, *p* = 0.790. Because the primary omnibus test was not significant, post hoc pairwise comparisons were not performed.

#### 3.4.3. Hardness Results

**Figure 8:**
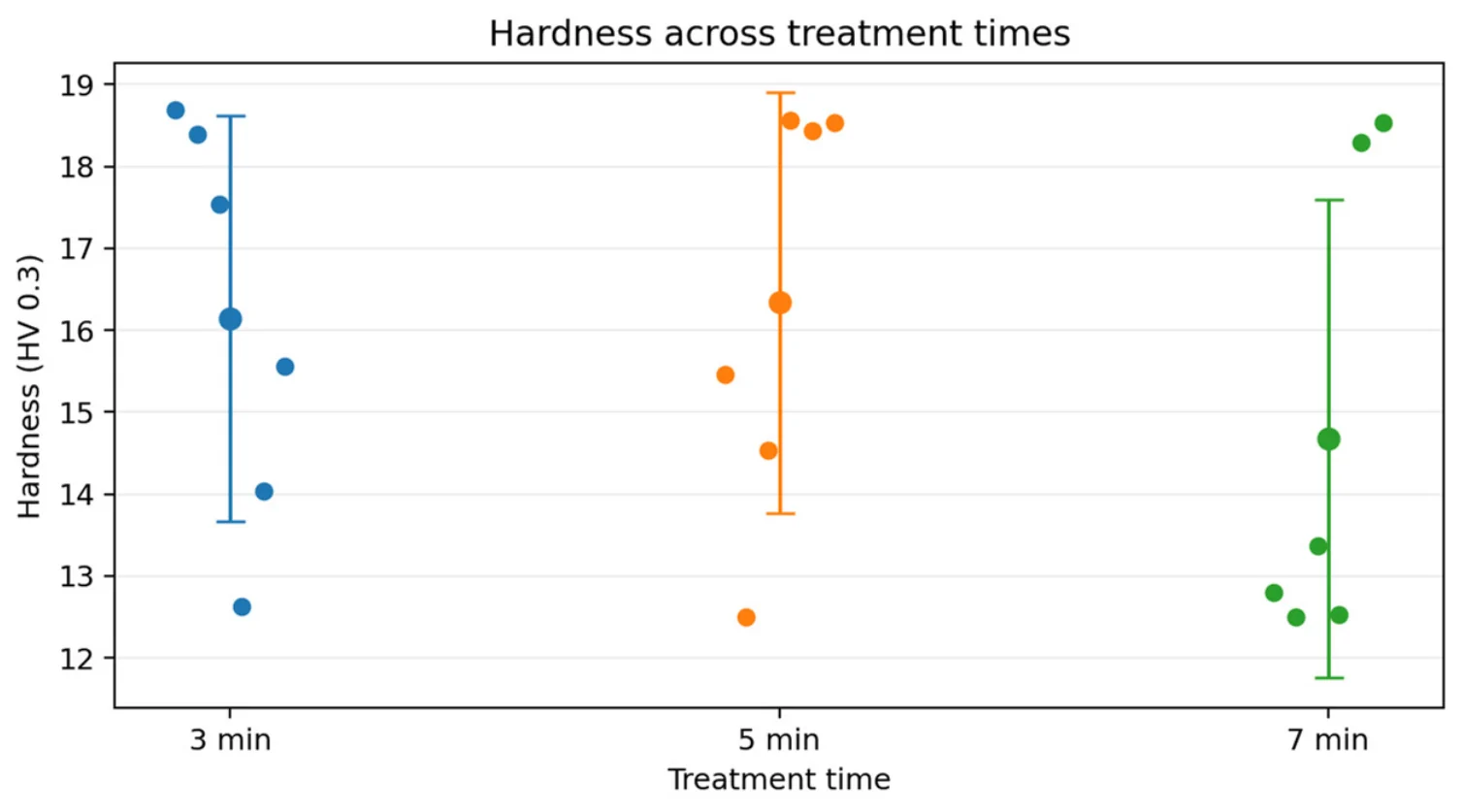
Hardness across treatment times different time intervals (3, 5, and 7 min). Points represent specimen-level values; the larger marker denotes the group mean and error bars represent ±1 SD.

Mean hardness was 16.144 ± 2.477 HV 0.3 at 3 min, 16.339 ± 2.566 HV 0.3 at 5 min, and 14.672 ± 2.918 HV 0.3 at 7 min, as shown in Figure 8. The one-way ANOVA did not identify a statistically significant difference among treatment durations, F(2, 15) = 0.704, *p* = 0.510, η^2^ = 0.086. Although the 7 min group had a lower mean, the difference was small relative to the variability among specimens and therefore was not statistically supported in this sample.

Welch’s ANOVA was consistent with the primary analysis, F(2, 9.95) = 0.598, *p* = 0.568. The conclusion therefore did not depend on the equal-variance assumption. No post hoc pairwise comparisons were performed because the omnibus ANOVA was non-significant. The complete one-way ANOVA results for both surface roughness and hardness is illustrated in Table 3.

## 4. Discussion

PMMA is the most popular denture base polymer‚ as it is relatively cost-effective and easy to work with, appears aesthetic‚ and has good biocompatibility and stability in the oral environment [27,28]. PMMA also has a number of disadvantages; the main disadvantage of which is its low mechanical strength, where it may fail as a denture due to repeated masticatory load or accidental dropping [3,27,28,29]. The fiber or mesh reinforcement of PMMA has been investigated for improving the mechanical properties of PMMA-based materials, and more recent developments have demonstrated the potentially beneficial role of NPs on these properties [28,29].

While several types of NPs have been studied‚ TiO_2_ has led to excellent outcomes due to its biocompatibility‚ chemical stability‚ non-toxicity‚ corrosion resistance‚ high strength, and intrinsic antimicrobial activity [27]. It was found to consistently increase the nanomechanical hardness and modulus of PMMA denture base systems and reduce bacterial adhesion [3]. A systematic review also concluded that the antimicrobial activity and surface hardness improved with TiO_2_ addition, largely irrespective of the particle size [7].

The efficacy of any nanofiller is highly dependent on its dispersion in the polymer matrix. Due to their high surface area and surface energy‚ NPs have a tendency to agglomerate or clump. These agglomerates can act as stress concentrations‚ resulting in a non-homogeneous stress distribution‚ and subsequent degradation of the mechanical performance [29]. Uniform dispersion and impregnation is therefore crucial to prevent the local stress concentrations and failure of the resin [7,29]. Recent investigations of nanocomposites have shown that the dispersion state of the NPs can be a stronger factor for mechanical reinforcement than the NP–polymer interaction strength [30].

Also‚ the referenced literature in dental materials showed that the incorporation of TiO_2_ uniformly distributed into acrylic resin has been shown to considerably improve tensile and impact strength; this is supported by SEM studies which demonstrated the best mechanical properties to correlate with the most homogenous composite mix (the experimental 1 wt% TiO_2_ nanocomposite had less micro-pores and cracks than that of the more loaded groups) [31,32]. However‚ beyond the threshold of filler agglomeration‚ the mechanical properties will decrease as a result of stress concentration in the aggregates that weakens the matrix [27,29]. This has also been reported for 3D-printed TiO_2_ nanocomposites‚ with increasing agglomeration and reduced flexural and impact resistance being observed at higher filler concentrations in SEM micrographs of the samples [27].

These are relevant to the current 3D print-based study‚ as 3D-printed denture base resins have been reported to have lower flexural strength‚ flexural modulus‚ impact strength and hardness than heat-polymerized PMMA [27,33]. One method of closing this gap is incorporating NPs, particularly ZrO_2_ NPs, which has been shown to increase flexural strength‚ impact strength‚ hardness and tensile strength of 3D-printed denture base resin‚ with the increase in properties likely due to the dispersal of the nano-filler in the polymer matrix [33,34]. For this 3D-printed ZrO_2_ system‚ agglomeration again acted as a stress concentration that promoted the initiation of cracks [34]. This further reiterates that whatever the type of nanofiller‚ their presence will only benefit reinforcement if they are well-dispersed throughout the matrix.

In the present study, TiO_2_ NPs were incorporated into a 3D-printed denture base resin and dispersed using probe sonication at three different time points (3‚ 5‚ and 7 min). Dispersion of TiO_2_ NPs in denture base resin was evaluated using SEM imaging‚ EDS mapping‚ and ATR-FTIR.

The clustering of the 3 min group indicates that this treatment was not long enough to fully disperse the aggregates with ultrasound. This is expected from the principle of ultrasonic dispersion; dispersion via sonication‚ which involves the formation of cavitations‚ is preferred to ball milling or high-shear mixing for redispersion of nanopowders because it can effectively break apart agglomerates, and longer or more energetic treatments yield more cavitation energy and this higher input can overcome the attractive forces binding particles in aggregates [35]. While a relatively long time window from 3 to 7 min provides a cumulative effect on the cavitation‚ an excessive sonication time will change the particles’ physicochemical properties and lead to sonication-generated agglomerates; thus, it is not necessarily expected that ultrasonication becomes more effective as time passes and it can be expected that there is a preferable range of time [35].

The 5 min group showed intermediate clustering and some agglomerates‚ and the 7 min group showed the most qualitatively uniform distribution and titanium map.

Additionally, the FTIR spectra of the three samples were similar‚ containing the same bands of the resin matrix‚ indicating that no gross difference in the detected functional-group profile was observed with respect to the sonication time‚ and that sonication was a means to improve the distribution of the TiO_2_ in the matrix‚ without altering its primary chemical structure.

It can therefore be assumed, according to the characterization studies, that the 7 min group dispersion was the more preferable out of the three groups‚ since it led to a more uniform microstructure and more homogeneous elemental distribution‚ both of which are relevant to the reinforcing role of the nanofiller. However, because dispersion was characterized only by morphology and elemental analysis‚ and EDS point values are only semi-quantitative and region-dependent, they are not representative of the clinical performance of the specimens. Therefore, surface and mechanical tests were additionally performed.

Surface roughness did not differ significantly among the three groups (*p* = 0.686), and had mean Ra values of 0.687, 0.835, and 0.701 μm at 3, 5, and 7 min, respectively. In NP-reinforced PMMA, surface roughness is governed largely by filler agglomerates that protrude at the finished surface; Ra increases when nanofillers begin to agglomerate or the loading exceeds a low threshold, whereas well-dispersed particles at low concentration leave the surface essentially unchanged. Gad et al. reported that adding 0.5 wt.% NPs produced only a non-significant change in Ra, while higher concentrations significantly increased it [36], and Gopalakrishnan et al. similarly found surface roughness to scale with the amount of NP incorporated [37]. At the low, fixed 0.3 wt.% loading, few surface-breaking clusters were present in any group, so roughness remained low and statistically flat regardless of sonication time. The numerical peak at 5 min is consistent with the residual, detectable agglomerates seen in the 5 min group by SEM, but the difference did not reach significance based on the tested sample size.

The present findings do not demonstrate a direct correlation between dispersion quality and surface roughness; they show only that roughness remained statistically comparable while the observed particle distribution changed qualitatively.

Vickers microhardness also did not differ significantly among the sonication groups. The 3- and 5 min groups had similar mean hardness values ≈ 16.14 and 16.34, respectively. Whereas the 7 min group had a numerically lower mean ≈ 14.67. Because the overall group effect was not significant, this difference must be described as a numerical tendency rather than a demonstrated reduction in hardness.

There are two main possible mechanisms regarding the numerically lower mean in the 7 min group. However, because molecular weight, degree of conversion (DC), crosslink density, gel permeation chromatography, and rheology were not measured in this study, the following are offered as plausible, non-mutually exclusive hypotheses rather than demonstrated mechanisms.

The prolonged probe sonication can be associated with damaged resin/matrix precursor, as ultrasonic cavitation has been reported to degrade dissolved polymers through mechanochemical chain scission, since the energy released by imploding cavitation bubbles can fracture polymer chains [38]; this behavior has been demonstrated particularly for PMMA, where ultrasonic irradiation reduces molecular weight, without limiting the photopolymerization capacity of the reactive methacrylate groups [39]. A reduction in chain length and crosslink density in the cured matrix could also lower surface hardness.

A complementary contribution is cure quality; in a close analogous study—silanized TiO_2_ in a 3D-printed denture base—Vickers hardness tracked the DC, with lower DC corresponding to lower hardness [27]. Any process that disturbs monomer or lowers DC (for example, localized heating or gas entrainment during extended sonication) would therefore reduce hardness. Microvoids left by cavitation bubbles in the longer-sonicated specimens can act in the same manner. That the loss saturates by 5 min is consistent with an early-onset process rather than one that accumulates linearly with exposure.

The second hypothesis is associated with curing-light scattering through the optical behavior of TiO_2_. For light-cured resins‚ surface hardness is an indirect measure of DC; a higher DC indicates a higher degree of cross-linked network, which is harder than one containing linear chains and residual monomer [40]‚ and conversion by itself depends on sufficient curing light reaching the full depth of the specimen [41]. TiO_2_ is a powerful scatterer of the 385 nm curing light, and inorganic filler particles considerably reduce light transmission through resin; the addition of filler has been shown to cut the amount of light reaching the base of a specimen by as much as 92% [41]. Metal-oxide nanofillers reduce light transmittance and thus the degree of cure through light scattering [40]. Because the TiO_2_ concentration was fixed at 0.3 wt.% and the printing and post-curing protocols were standardized across groups, differences in total filler content or nominal curing exposure cannot explain the hardness pattern. Nevertheless, sonication-induced changes in particle distribution and agglomerate size could theoretically alter the interaction between TiO_2_ and the curing light. Filler particles can modify transmitted irradiance through light scattering, and adequate light delivery affects polymerization; however, the direction of this effect is material- and dispersion-dependent rather than uniformly detrimental [27,41].

The isolated ~5 μm crack seen in a 7 min SEM field is supportive of a locally weakened, over-sonicated matrix, where defects and agglomerates can act as stress-concentration sites at which cracks initiate [27]. However, a single crack under the electron beam is ambiguous: electron bombardment of polymers under vacuum can induce crack growth at stresses well below those required mechanically [42]. It should therefore be assessed cautiously as consistent with matrix damage, without absolute proof, and only featured if reproducible across multiple 7 min fields.

Taken together within the limitations of this study, the 7 min group showed the most qualitatively uniform TiO_2_ distribution by SEM/EDS. Vickers microhardness did not differ significantly among the sonication groups, although the 7 min group had a numerically lower mean than the 3- and 5 min groups. These findings suggest that, at the fixed TiO_2_ concentration and under the investigated processing conditions, sonication time primarily affected the observed physical distribution of the NPs without producing statistically detectable changes in the measured surface properties. Accordingly, the present data do not demonstrate a direct correlation between dispersion quality and either roughness or hardness; instead, they show that these outcomes can remain partly independent within the tested sonication range. The lower numerical hardness observed at 7 min warrants further investigation using direct DC and polymer-network analyses.

Several limitations moderate these interpretations; lack of quantitative SEM data (agglomerate number, mean/median size, area fraction, and size distribution) to reliably verify the most uniform dispersion; the use of only three specimens per group for roughness and hardness tests, for which a larger sample size is needed to confirm the non-significant roughness differences and the hardness trend; only a single fixed TiO_2_ loading (0.3 wt.%) and a single set of sonication-power settings were tested without pure resin (0 wt.% TiO_2_) or unsonicated modified control groups. As a result, the findings describe the impact of sonication time within this fixed system, not the effect of loading or amplitude. DC and molecular weight were not measured, and no flexural, impact, or fatigue testing was performed; the hardness and roughness results characterize surface behavior and cannot predict bulk mechanical performance without further testing.

## 5. Conclusions

Within the limits of this study‚ the probe-sonication time affected the TiO_2_ NPs dispersion and surface properties of the 3D-printed denture base resin at the same filler loading. Analysis by SEM and semi-quantitative EDS showed that with increasing probe-sonication time‚ the TiO_2_ NPs in the 3D-printed denture base resin exhibited improved dispersion and reduced agglomeration. The 7 min sonication group showed the most homogeneous microstructure and titanium distribution, although complete deagglomeration was not achieved in any group. The ATR-FTIR spectra showed no difference among the groups‚ indicating that sonication changed the physical distribution of the NPs in the resin matrix‚ without changing the functional groups of the resin matrix.

Surface characterization showed that the surface-mechanical properties did not improve with better dispersion; Vickers microhardness was numerically highest in the 5 min group, and lowest in the 7 min group, but these differences were not significant under the conditions studied. The surface roughness was also the highest in the 5 min group, and similarly the differences were not statistically significant.

Accordingly, 7 min may be considered the most suitable sonication duration for improving TiO_2_ distribution in this 3D-printed denture-base resin system. However, this recommendation is limited to the tested TiO_2_ loading and processing conditions and should not be interpreted as an overall optimum. Further investigations are needed to fully explain the effect of sonication time on the TiO_2_-3D printing resin performance. In the present study, neat-resin and unsonicated controls were not assessed; the degree of dispersion was only qualitatively evaluated; sample size for the mechanical tests was relatively small (*n* = 6); and DC and other mechanical tests such as flexural strength‚ elastic modulus‚ and impact strength were not performed. 

## Figures and Tables

**Table 1:** Diagnostic assessment of the one-way ANOVA models.

Outcome	Shapiro W	*p*	Levene F	*p*	Max |Studentized Residual|	Interpretation
Surface roughness	0.929	0.186	3.194	0.070	2.69	No clear overall violation detected
Hardness	0.909	0.083	0.304	0.742	1.59	No clear overall violation detected

**Table 2:** Specimen-level descriptive statistics by treatment time.

Outcome	Time	*n*	Mean	Standard Deviation (SD)	95% Confidence Interval for Mean
Surface roughness	3 min	6	0.687	0.209	0.467 to 0.906
Surface roughness	5 min	6	0.835	0.463	0.349 to 1.321
Surface roughness	7 min	6	0.701	0.231	0.458 to 0.944
Hardness (HV 0.3)	3 min	6	16.144	2.477	13.545 to 18.743
Hardness (HV 0.3)	5 min	6	16.339	2.566	13.646 to 19.032
Hardness (HV 0.3)	7 min	6	14.672	2.918	11.610 to 17.734

*n* is the number of independent specimens. Each group value is the mean of the averaged three technical readings of each specimen.

**Table 3:** Complete one-way ANOVA results for treatment-time effects.

Outcome	Source	SS	df	MS	F	*p*	η^2^
Surface roughness	Between groups	0.080	2	0.040	0.386	0.686	0.049
	Within groups	1.560	15	0.104			
	Total	1.640	17				
Hardness	Between groups	9.966	2	4.983	0.704	0.510	0.086
	Within groups	106.161	15	7.077			
	Total	116.127	17				

## Data Availability

The raw data supporting the conclusions of this article will be made available by the authors on request.

## Supplementary Materials

The following supporting information can be downloaded at https://www.mdpi.com/article/10.3390/polym18192407/s1, Figure S1: SEM-EDS Images of the 3 min probe sonicated samples: (a) SE-SEM surface image showing particle distribution; and corresponding elemental maps of (b) carbon, (c) oxygen, and (d) silicone; Figure S2: SEM-EDS Images of the 5 min probe sonicated samples: (a) SE-SEM surface image showing particle distribution; and corresponding elemental maps of (b) carbon, (c) oxygen, and (d) silicone; Figure S3: SEM-EDS Images of the 7 min probe sonicated samples: (a) SE-SEM surface image showing particle distribution; and corresponding elemental maps of (b) carbon, (c) oxygen, and (d) silicone; Figure S4: Normal Q-Q plot of residuals from the surface roughness ANOVA model; Figure S5: Normal Q-Q plot of residuals from the hardness ANOVA model.

## Abbreviations

The following abbreviations are used in this manuscript:

PMMA	Polymethyl methacrylate
CAD/CAM	Computer-aided design/computer-aided manufacturing
3D	Three-dimensional
NP	Nanoparticle
TiO_2_	Titanium dioxide
SEM	Scanning electron microscopy
TEM	Transmission electron microscopy
EDS	Energy-dispersive X-ray spectroscopy
ATR-FTIR	Attenuated total reflectance-Fourier transform infrared
FTIR	Fourier transform infrared
SD	Standard deviation
DC	Degree of conversion

## References

[1] Sabouhi M., Amini-Pozveh M., Bajoghli F., reza Dastjerd H., Mohammadi R. (2022). Synthesis and characterization of antifungal nanocomposite AgSiO_2_ polymethyl methacrylate. Eur. J. Dent..

[2] AlGhamdi M.A., Fouda S.M., Taymour N., Akhtar S., Khan S.Q., Ali M.S., Elakel A.M., Nassar E.A., Gad M.M. (2023). Comparative Evaluation of TiO_2_ Nanoparticle Addition and Postcuring Time on the Flexural Properties and Hardness of Additively Fabricated Denture Base Resins. Nanomaterials.

[3] Alrahlah A., Fouad H., Hashem M., Niazy A.A., AlBadah A. (2018). Titanium oxide (TiO_2_)/polymethylmethacrylate (PMMA) denture base nanocomposites: Mechanical, viscoelastic and antibacterial behavior. Materials.

[4] Azab A., Abdelhady W.A., Elwakeel E., Ashraf M., Wally R., Soliman A., Mohamed M.A., Abozaid D. (2025). Systematic review and meta analysis of mechanical properties of 3D printed denture bases compared to milled and conventional materials. Sci. Rep..

[5] Alharbi N., Osman R., Wismeijer D. (2016). Effects of build direction on the mechanical properties of 3D-printed complete coverage interim dental restorations. J. Prosthet. Dent..

[6] Bento V.A.A., Gomes J.M.d.L., Oliveira-Limirio J.P.J., Rosa C.D.D.R.D., Lemos C.A.A., dos Santos D.M., Pellizzer E.P. (2024). Effect of Aging on the Mechanical Properties of CAD/CAM-Milled and 3D-Printed Acrylic Resins for Denture Bases. Int. J. Prosthodont..

[7] Kaurani P., Hindocha A.D., Jayasinghe R.M., Pai U.Y., Batra K., Price C. (2023). Effect of addition of titanium dioxide nanoparticles on the antimicrobial properties, surface roughness and surface hardness of polymethyl methacrylate: A Systematic Review. F1000Research.

[8] Sun J., Forster A.M., Johnson P.M., Eidelman N., Quinn G., Schumacher G., Zhang X., Wu W.-l. (2011). Improving performance of dental resins by adding titanium dioxide nanoparticles. Dent. Mater..

[9] Zaragoza-Contreras E.A., Hernández-Escobar C.A., Mendoza-Duarte M.E., Flores-Gallardo S.G., Ibarra-Gómez R., Márquez-Lucero A. (2009). Thermal and Mechanical Analysis of Silver/Carbon Nanoparticle—PMMA Nanocomposites Obtained by Miniemulsion Polymerization. Polym. J..

[10] Hielscher K. (2012). Ultrasonic Milling and Dispersing Technology for Nano-Particles. MRS Proc..

[11] Chu C.-Y., Li Y.-C., Jang G.-W., Pu Y.-C., Chen M.-Z., Chen P.-Y. (2019). Qualitative Effect of the Polymerization Rate on the Nanoparticle Dispersion in Poly(methyl methacrylate)/Silica Nanocomposite Films. Macromolecules.

[12] Salman A.D., Jani G.H., Fatalla A.A. (2017). Comparative study of the effect of incorporating SiO_2_ nano-particles on properties of poly methyl methacrylate denture bases. Biomed. Pharmacol. J..

[13] Wu W., Ichihara G., Suzuki Y., Izuoka K., Oikawa-Tada S., Chang J., Sakai K., Miyazawa K., Porter D., Castranova V. (2014). Dispersion Method for Safety Research on Manufactured Nanomaterials. Ind. Health.

[14] Windey R., AhmadvashAghbash S., Soete J., Swolfs Y., Wevers M. (2023). Ultrasonication optimisation and microstructural characterisation for 3D nanoparticle dispersion in thermoplastic and thermosetting polymers. Compos. Part B Eng..

[15] Hang P.T.T. (2024). Investigating ultrasonic dispersion of nanostructured silica for nanocomposite materials application. Univ. Danang-J. Sci. Technol..

[16] Jokar A., Azizi M., Hamidi Esfahani Z. (2015). Effects of ultrasound time on the properties of polyvinyl alcohol-based nanocomposite films. Nutr. Food Sci. Res..

[17] Montazeri A., Khavandi A., Javadpour J., Tcharkhtchi A. (2011). An Investigation on the Effect of Sonication Time and Dispersing Medium on the Mechanical Properties of MWCNT/Epoxy Nanocomposites. Adv. Mater. Res..

[18] Park S.S., Bernet N., de la Roche S., Hahn H.T. (2003). Processing of Iron Oxide-epoxy Vinyl Ester Nanocomposites. J. Compos. Mater..

[19] Iyer Ganapathi J., Lee S.S., Kalyon D.M., Fisher F.T. (2020). Impact of ultrasonication on carbon nanotube demixing and damage in polymer nanocomposites. J. Appl. Polym. Sci..

[20] Qi J., Ye Y.Y., Wu J.J., Wang H.T., Li F.T. (2013). Dispersion and stability of titanium dioxide nanoparticles in aqueous suspension: Effects of ultrasonication and concentration. Water Sci. Technol..

[21] Bittmann B., Haupert F., Schlarb A.K. (2009). Ultrasonic dispersion of inorganic nanoparticles in epoxy resin. Ultrason. Sonochem..

[22] Nguyen T.-C., Nguyen T.-D., Vu D.-T., Dinh D.-P., Nguyen A.-H., Ly T.-N.-L., Dao P.-H., Nguyen T.-L., Bach L.-G., Thai H. (2020). Modification of Titanium Dioxide Nanoparticles with 3-(Trimethoxysilyl)propyl Methacrylate Silane Coupling Agent. J. Chem..

[23] Chaijareenont P., Takahashi H., Nishiyama N., Arksornnukit M. (2012). Effect of different amounts of 3-methacryloxypropyltrimethoxysilane on the flexural properties and wear resistance of alumina reinforced PMMA. Dent. Mater. J..

[24] Waghmare A., Nair C., Shukla A.K., Chaturvedi M., Bhagat T.V., Alsubaiy E.F., Suleman G., Khader M.A., Chaturvedi S. (2024). Flexural strength and surface hardness of nanocomposite denture base resins. Heliyon.

[25] Cierech M., Szerszeń M., Wojnarowicz J., Łojkowski W., Kostrzewa-Janicka J., Mierzwińska-Nastalska E. (2020). Preparation and Characterisation of Poly(methyl metacrylate)-Titanium Dioxide Nanocomposites for Denture Bases. Polymers.

[26] Alwan S.A., Alameer S.S. (2015). The effect of the addition of silanized Nano titania fillers on some physical and mechanical properties of heat cured acrylic denture base materials. J. Baghdad Coll. Dent..

[27] Altarazi A., Haider J., Alhotan A., Silikas N., Devlin H. (2023). 3D printed denture base material: The effect of incorporating TiO_2_ nanoparticles and artificial ageing on the physical and mechanical properties. Dent. Mater..

[28] Gad M.M., Fouda S.M., Al-Harbi F.A., Napankangas R., Raustia A. (2017). PMMA denture base material enhancement: A review of fiber, filler, and nanofiller addition. Int. J. Nanomed..

[29] Alhotan A., Yates J., Zidan S., Haider J., Silikas N. (2021). Assessing Fracture Toughness and Impact Strength of PMMA Reinforced with Nano-Particles and Fibre as Advanced Denture Base Materials. Materials.

[30] Colijn I., Postma E., Fix R., van der Kooij H.M., Schroën K. (2024). Particle dispersion governs nano to bulk dynamics for tailored nanocomposite design. J. Colloid Interface Sci..

[31] Ghahremani L., Shirkavand S., Akbari F., Sabzikari N. (2017). Tensile strength and impact strength of color modified acrylic resin reinforced with titanium dioxide nanoparticles. J. Clin. Exp. Dent..

[32] Moslehifard E., Robati Anaraki M., Shirkavand S. (2019). Effect of adding TiO_2_ nanoparticles on the SEM morphology and mechanical properties of conventional heat-cured acrylic resin. J. Dent. Res. Dent. Clin. Dent. Prospects.

[33] Alshaikh A.A., Khattar A., Almindil I.A., Alsaif M.H., Akhtar S., Khan S.Q., Gad M.M. (2022). 3D-Printed Nanocomposite Denture-Base Resins: Effect of ZrO_2_ Nanoparticles on the Mechanical and Surface Properties In Vitro. Nanomaterials.

[34] Al-Sammraaie M.F., Fatalla A.A., Atarchi Z.R. (2024). Assessment of the correlation between the tensile and diametrical compression strengths of 3D-printed denture base resin reinforced with ZrO_2_ nanoparticles. J. Baghdad Coll. Dent..

[35] Glaubitz C., Rothen-Rutishauser B., Lattuada M., Balog S., Petri-Fink A. (2022). Designing the ultrasonic treatment of nanoparticle-dispersions via machine learning. Nanoscale.

[36] Gad M.M., Abualsaud R., Al-Thobity A.M., Baba N.Z., Al-Harbi F.A. (2020). Influence of Addition of Different Nanoparticles on the Surface Properties of Poly(methylmethacrylate) Denture Base Material. J. Prosthodont..

[37] Thomas S., Gopalakrishnan S., Kalazikkal N. (2018). OSC21: Morphological, Mechanical and Biological Properties of Silver Nanoparticle Decorated Denture Base Polymer. J. Indian Prosthodont. Soc..

[38] Doulah M.S. (1978). A proposed mechanism for the degradation of addition polymers in cavitating ultrasonic fields. J. Appl. Polym. Sci..

[39] Wu C.B., Sheth P.J., Johnson J.F. (1977). Ultrasonic degradation of poly(methyl methacrylate. Polymer.

[40] Dutra Borges B.C., Groninger A.I.S., Soares G.P. (2011). Curing Quality of Composites as Influenced by the Filler Content, Light Source and Curing Time. Dentistry.

[41] Faria-e-Silva A.L., Pfeifer C.S. (2017). Impact of thio-urethane additive and filler type on light-transmission and depth of polymerization of dental composites. Dent. Mater..

[42] Dickinson J.T., Klakken M.L., Miles M.H., Jensen L.C. (1985). Electron-beam-induced fracture of polymers. J. Polym. Sci. Polym. Phys. Ed..

