# Supplementary material for: Time-Dependent Dispersion of TiO2 Nanoparticles in 3D-Printed Denture Base Resin Using Probe Sonication
Source: Polymers (Basel). 2026 Oct 2;18(19):2407. doi: 10.3390/polym18192407 (PMC13666636; doi:10.3390/polym18192407)
Supplement: Supplementary file 1 [file polymers-18-02407-s001.zip › polymers-4531193-supplementary.pdf]

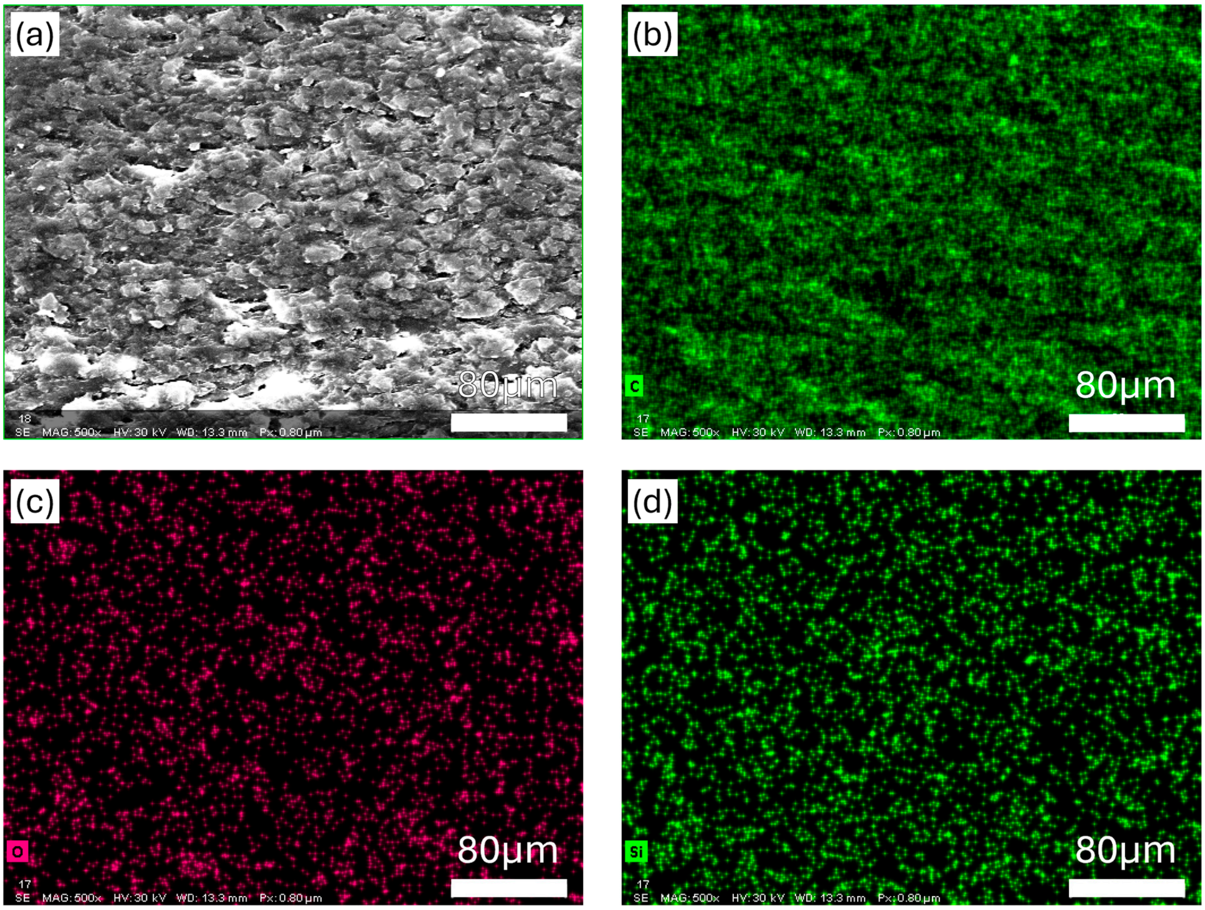

Figure S1. SEM-EDS Images of the 3-minute probe sonicated samples: **(a)** SE-SEM surface image showing particle distribution; and corresponding elemental maps of **(b)** carbon, **(c)** oxygen, and **(d)** silicone.

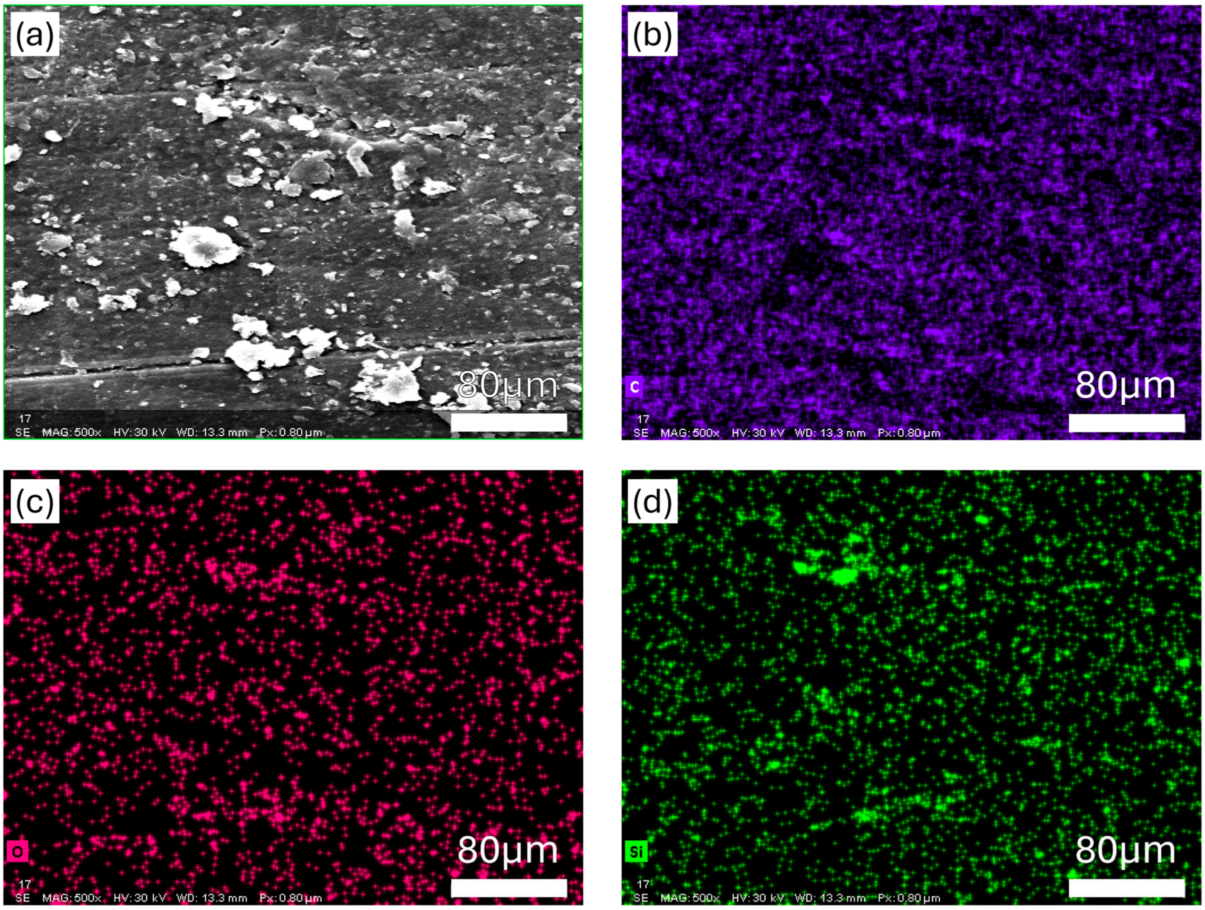

Figure S2. SEM-EDS Images of the 5-minute probe sonicated samples: **(a)** SE-SEM surface image showing particle distribution; and corresponding elemental maps of **(b)** carbon, **(c)** oxygen, and **(d)** silicone.

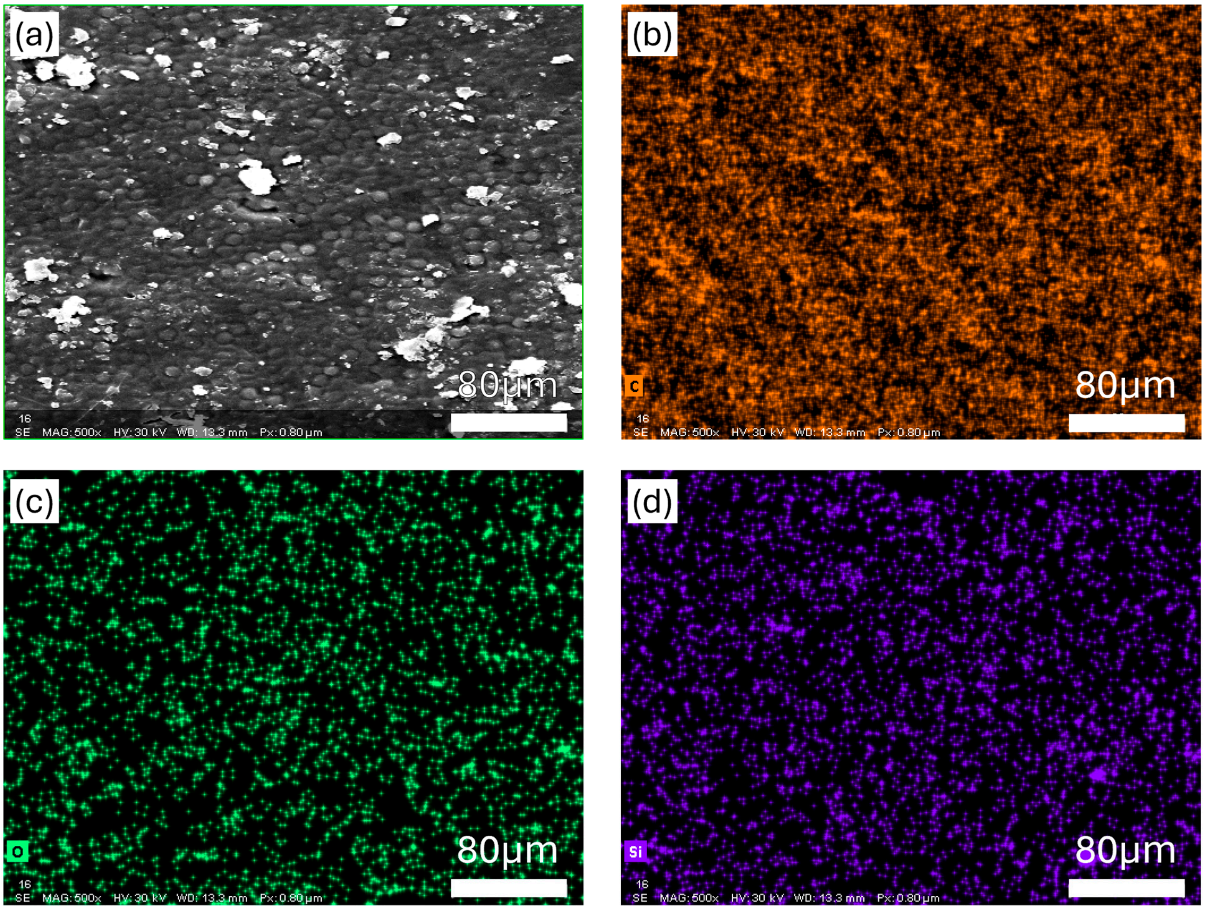

Figure S3. SEM-EDS Images of the 7-minute probe sonicated samples: **(a)** SE-SEM surface image showing particle distribution; and corresponding elemental maps of **(b)** carbon, **(c)** oxygen, and **(d)** silicone.

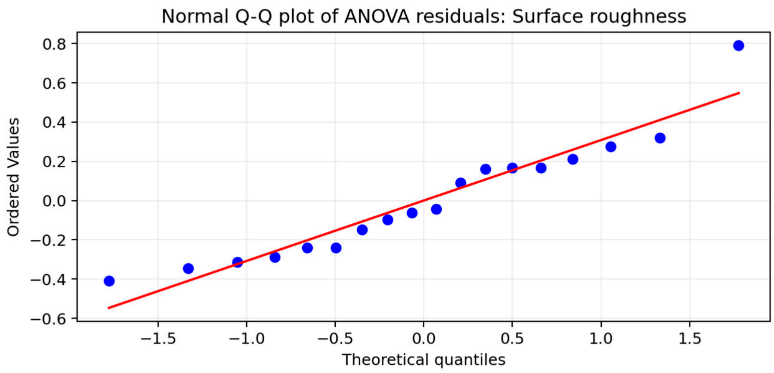

Figure S4. Normal Q-Q plot of residuals from the surface roughness ANOVA model.

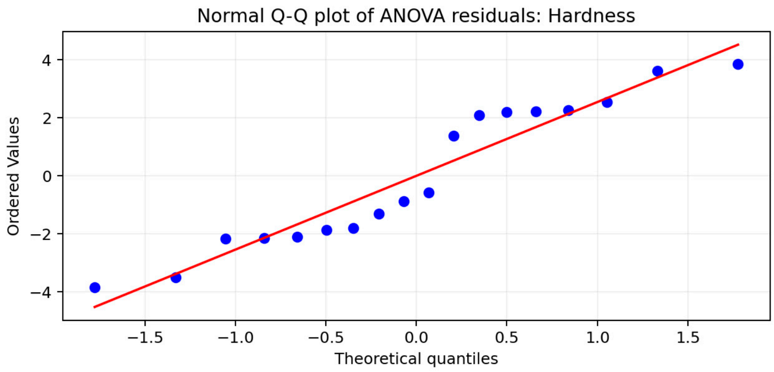

Figure S5. Normal Q-Q plot of residuals from the hardness ANOVA model.
